# Cyanobacterial Community Composition and Bacteria–Bacteria Interactions Promote the Stable Occurrence of Particle-Associated Bacteria

**DOI:** 10.3389/fmicb.2018.00777

**Published:** 2018-04-26

**Authors:** Jason N. Woodhouse, Jennifer Ziegler, Hans-Peter Grossart, Brett A. Neilan

**Affiliations:** ^1^Department of Experimental Limnology, Leibniz-Institute of Freshwater Ecology and Inland Fisheries, Berlin, Germany; ^2^School of Biotechnology and Biomolecular Sciences, University of New South Wales, Sydney, NSW, Australia; ^3^School of Environmental and Life Sciences, The University of Newcastle, Callaghan, NSW, Australia

**Keywords:** cyanobacteria, microbe-interactions, microcystin, particle-associated, co-occurrence analysis

## Abstract

Within meso/eutrophic freshwater ecosystems the dominance of cyanobacterial blooms during summer months has substantial impacts on ecosystem function with the production of toxins and subsequent induction of hypoxia altering food web structures and biogeochemical cycles. Cyanobacterial aggregates are extensively colonized by heterotrophic bacteria that provide the cyanobacteria with key nutrients and contribute towards remineralisation of organic matter. Here we sampled from five sites within a shallow eutrophic pond over a 6 months period, relating changes in the abundance of particle-associated heterotrophic taxa to phytoplankton abundance, toxin gene copies and physiochemical properties. The abundance of a majority of particle-associated bacteria were stable, in that they persisted despite perturbation. Cyanobacterial species abundance more likely correlated with stable rather than unstable bacteria and unstable bacteria were associated with allochthonous (terrestrial) organic matter. The occurrence of the most stable bacteria was correlated with large numbers of other bacteria suggesting bacteria-bacteria interactions have implications for the stable occurrence of microorganisms on particles. Freshwater ecosystems are frequently inundated with fresh nutrients in the form of surface runoff and experience an increasing number of high temperature days. In addition to increasing the severity and longevity of cyanobacterial blooms, run-off changes the nature of the particle-associated community compromising stability. This disruption has the potential to drive changes in the carbon and nitrogen cycles and requires further attention.

## Introduction

Cyanobacterial blooms are becoming increasingly common in freshwater ecosystems in response to a changing climate. Increasing temperatures, modified hydrology and nutrient enrichment result in substantial effects on microbial biomass production and community composition in freshwater systems (Carey et al., 2011). The quality of water is strongly influenced by anthropogenic activities, which lead to hypertrophication, especially with phosphorus and nitrogen. Due to the formation of large scums on the surface of the water bodies, the penetration of light into the deeper layers is reduced. This can lead to a depletion of oxygen affecting overall ecosystem functioning. Another risk of blooms is the release of toxins produced by certain cyanobacterial species, which can be harmful to human and animal health. Numerous heterotrophic bacteria are associated with cyanobacteria and have a substantial influence on cyanobacterial growth (Eiler and Bertilsson, 2004; Kolmonen et al., 2004; Tuomainen et al., 2006; Dziallas and Grossart, 2011; Klawonn et al., 2015; Adam et al., 2016). Cyanobacteria are often associated with different types of heterotrophic bacteria, which use the carbon fixed by photoautotrophs and play an essential role in the aquatic food web (Azam et al., 1983; Pomeroy and Wiebe, 1988; Grossart, 2010; Woodhouse et al., 2016).

The taxonomic distribution and the frequency of cyanobacterial-heterotrophic bacterial associations can be influenced by abiotic factors including nutrients, light and temperature conditions, pH and salinity as well as biotic factors like grazing (Dziallas and Grossart, 2011, 2012). Cyanobacteria, in turn, can influence the environment through metabolic activities such as photosynthesis (Paerl, 2000; Wilson et al., 2006; Havens, 2008). In this process cyanobacterial cells generate oxygen, cause a rise in pH and exude a number of small organic molecules and nutrients including ammonia (Klawonn et al., 2015).

Furthermore, colonial and filamentous cyanobacteria offer microenvironments in which interactions between bacteria and cyanobacteria take place (Salomon et al., 2003; Dziallas and Grossart, 2012; Klawonn et al., 2015; Adam et al., 2016; Briand et al., 2016). In this habitat, “the phycosphere” (Bell and Mitchell, 1972), positive or negative interactions between cyanobacteria and their associated bacteria can occur (Woodhouse et al., 2016). It has been reported that during an algal bloom the abundance and the amounts of heterotrophic bacteria are substantially affected by the cell concentration of cyanobacteria (Bouvy et al., 2001; Eiler and Bertilsson, 2004). By exudation or cell lysis it is possible for cyanobacteria to release diverse types of organic molecules like ammonium, carbohydrates, lipids, proteins and organic acids but also bioactive compounds such as toxins (Amemiya et al., 1990; Sivonen and Börner, 2008; Tonietto et al., 2014; Kehr and Dittmann, 2015; Adam et al., 2016).

Temporal dynamics are a major part of aquatic ecosystems with short and long term shifts in microbial community composition manifesting to different extents. Seasonal changes in microbial composition and activity in a temperate environment are strongly affected by physical parameters, most notably temperature, which has a direct effect on the activity, species composition and density of bacteria and bacterivore assemblages (Caron et al., 1986; Pomeroy et al., 1991; Dziallas and Grossart, 2011). Short-term changes (hours to days) likely manifest as a consequence of biological features of the community, such as with predator-prey relationships, where rapid oscillations in the abundance of bacteria and their consumers are evident. Transient storm events also may significantly affect microbial processes on short time scales (Wikner et al., 1990). Understanding under which conditions the occurrence of freshwater bacteria are stable is critical for understanding the individual and cumulative impact of bacteria on biogeochemical processes. With this study we sought to test the hypothesis that constant changes in the cyanobacterial community composition, and influx of allochthonous organic matter, would reduce the stable-occurrence of particle-associated heterotrophic bacteria. By assessing the connectivity and stable occurrence of particle associated bacteria across this 6 months period, we highlight differences in stability across freshwater phyla and demonstrate how stability relates to changing cyanobacterial species composition and interactions between heterotrophic bacteria.

## Materials and Methods

### Site Description and Sampling

Water samples were collected from five sites within Kensington Pond, Centennial Park, 4 km southeast of the Sydney CBD. Collection of samples was performed in conjunction with the NSW Office of Water from December 2014 to April 2015. Measurement of *in situ* environmental parameters [conductivity, temperature, dissolved oxygen, fluorescent dissolved organic matter (fDOM), pH, chlorophyll, Phycocyanin, and turbidity] were performed using a Hydrolab DS5 water quality sonde. From each site duplicate 1,000 ml and a single 250 ml sub-surface water samples were collected at a depth of 0.25 m. The 250 ml samples were preserved using 3 ml of Lugol’s iodine solution and sent to the NSW Office of Water for calculation of cell counts and biovolumes. Biovolumes were estimated using a reference list of cell dimensions compiled from multiple sources (e.g., Newcombe, 2009), as previously described (Hötzel and Croome, 1999). Each 1,000 ml sample was immediately transported back to the laboratory and filtered within 3 h of the initial sampling on a 3 μm Millipore^TM^ GSWP membrane. Filters were then stored in RNA later at -80°C.

### DNA Extraction and Sequencing

DNA extraction was performed using a protocol modified from Morin et al. (2010). Briefly filters were removed from RNA later, dissected into quarters and placed in 50 ml tubes containing TE buffer (10 mM Tris, 1 mM EDTA, pH 8.0). Filters were subjected to three freeze-thaw cycles between liquid nitrogen and a 30°C water bath. Between each cycle filters were vortexed briefly to dislodge adhered cells. Visual inspection of the pre-lysate indicated resuspension of a significant portion of biomass as indicated by the strong green color. Lysozyme (final concentration 10 mg ml^-1^) was added to this suspension, which was incubated at 37°C for 1 h. Proteinase K (1 mg ml^-1^) and SDS (final concentration 1% w/v) were then added and the suspension incubated a further 1 h at 55°C. Removal of carbohydrates was achieved by addition of CTAB (final conc. 1% w/v) and NaCl (final conc. 0.5 M), followed by incubation at 65°C for 10 min. Phase separation was performed using 1 volume chloroform/isoamyl alcohol and nucleic acids precipitated by the addition of 0.6 volume of isopropanol. DNA pellets were washed with 70% w/v ice-cold ethanol, air-dried and resuspended in TE buffer. High purity DNA was achieved by subsequently extracting DNA with the Genomic DNA Clean and Concentrator^TM^-10 kit (Zymo Research, Irvine, CA, United States).

The V1-3 region of the 16S rRNA gene was amplified using primers 27f and 515r using Velocity DNA polymerase (Bioline Pty Ltd., Sydney, NSW, Australia) as previously described (Woodhouse et al., 2016). Equimolar pooling was performed using the SequalPrep 96 well plate kit. Amplicon sequencing (2 × 300 bp PE) was performed at the Ramaciotti Centre for Genomics, the University of New South Wales (UNSW), Australia using the Miseq^TM^ (Illumina Inc., San Diego, CA, United States) instrument. De-multiplexed sequences are available for download via the NCBI short read archive under BioProject PRJNA448770. Quantitative PCR of the cyanobacterial 16S rRNA gene as well as genes for the biosynthesis of saxitoxin, microcystins, and cylindrospermopsin was performed using the Phytoxigene^TM^ CyanoDTec qPCR assay (Diagnostic Technology, Belrose, NSW, Australia). We generated calibration curves using the CyanoNAS set of verified standards, certified by the National Measurement Institute, Australia. PCR reactions were prepared and quantification undertaken using a Cepheid SmartCycler (Cepheid Inc., Sunnyvale, CA, United States) as previously described (Crawford et al., 2017).

### Bioinformatic and Statistical Analyses

Sequences were generated from the 5′ end of each 16S primer (27f and 519r) ensuring that the primers were not included in the final reads. Furthermore, sequences were obtained from the sequencing facility following de-multiplexing allowing for a single mismatch per barcode. Additional screening of sequences, including removal of erroneous or ambiguous bases, removal of chimeric sequences, contiguous assembly and filtering was performed using Mothur v. 1.39.5 (Schloss et al., 2009). OTU clustering was performed using the average neighbor method (Schloss and Westcott, 2011) at a distance of 0.03. Taxonomic assignment was performed using a combination of the Greengenes and Freshwater Microbial Field Guide (Newton et al., 2011) databases^1^ and the 16S Taxonomic Assignment Workflow^2^. Samples, including cyanobacterial reads were sub-sampled at the minimum sequencing depth of 24,000 sequences per sample. For each sample the absolute abundance of cyanobacteria was determined by cell counts. This value was then used to convert relative abundances of all particle-associated bacteria to absolute abundances by applying a correction factor based on the relative abundance of cyanobacteria within in each sample. For instance, a sample containing 1,000 cyanobacterial cells per ml and 50% cyanobacterial sequences was estimated to contain a total of 2,000 bacterial cells per ml, with the remaining 1,000 cells distributed amongst the non-cyanobacterial population. We acknowledge that this method is not preferred to cell counts of particle-associated bacteria using light microscopy (Porter and Feig, 1980), and suffers due to differences in gene copy numbers, but estimated absolute counts are inherently less flawed than relative abundances for analysis where we seek to relate bacterial abundances with environmental factors and cell counts (Dziallas and Grossart, 2011; Eiler et al., 2012; Peura et al., 2015; Woodhouse et al., 2016). Following this correction, all cyanobacterial reads were removed from the analysis. Alpha and beta diversity were inferred using PRIMER 6 (Clarke and Warwick, 2005).

Non-abundant OTUs, those that never exceeded 0.1% in a single sample, were removed from the analysis as their presence, a primary determinant of stability is questionable at such low abundances. The remaining dataset comprised 75.3±9.2% of bacterial sequences, with the decrease largely explained by samples in the latter stages of the sampling period. For the remaining OTUs stability, referring to the persistence of individual OTUs across the sampling period, was estimated using the coefficient of variation (CV) as calculated from the absolute abundances of OTUs. The CV is a dimensionless representation of the standard deviation, as determined only when a read was observed, accounting for the mean abundance within the dataset and allows for comparison between highly abundant and less abundant OTUs. The CV is typically a continuum meaning each OTU is considered more or less stable than each other OTU. Where the CV value exceeds 200, the observed abundance of an OTU exceeds two standard deviations or the 95% CI of its mean and as such as considered instable. OTUs with fewer than five observations possessed artificially low CV values due to the high number of zero values. We instead considered these to be part of the instable community as their occurrence was sporadic. (Linz et al., 2017).

Pairwise Spearman correlations between absolute abundances of abundant OTUs, environmental data and cyanobacterial biovolumes were calculated using the rcorr function in the Hmisc v. 4.0-2 library in R 1.0.136 and following false-discovery rate correction, were visualized as a network using Cytoscape v. 3.4.0 (Shannon et al., 2003; Smoot et al., 2011). For the resulting network we derived a set of metrics for each variable (OTUs, environmental data and biovolumes). Specifically, we considered the degree (number of correlations for each individual), the clustering coefficient (proportion of potential correlations realized by neighboring nodes), and betweenness centrality (proportion of passes through a variable for each shortest path between any two variables). Each of the three variables can be considered along an ecological spectrum with degree representing the number of potential direct effects, clustering coefficient representing the number of indirect effects, and the betweenness centrality reflecting the importance of control over all nodes within the network. Several studies have highlighted degree or betweenness centrality as potential proxies for identifying keystone species whose responses to external perturbations dictate community responses (Eldridge et al., 2015; Guidi et al., 2016).

## Results

### Community Composition

**FIGURE 1:**
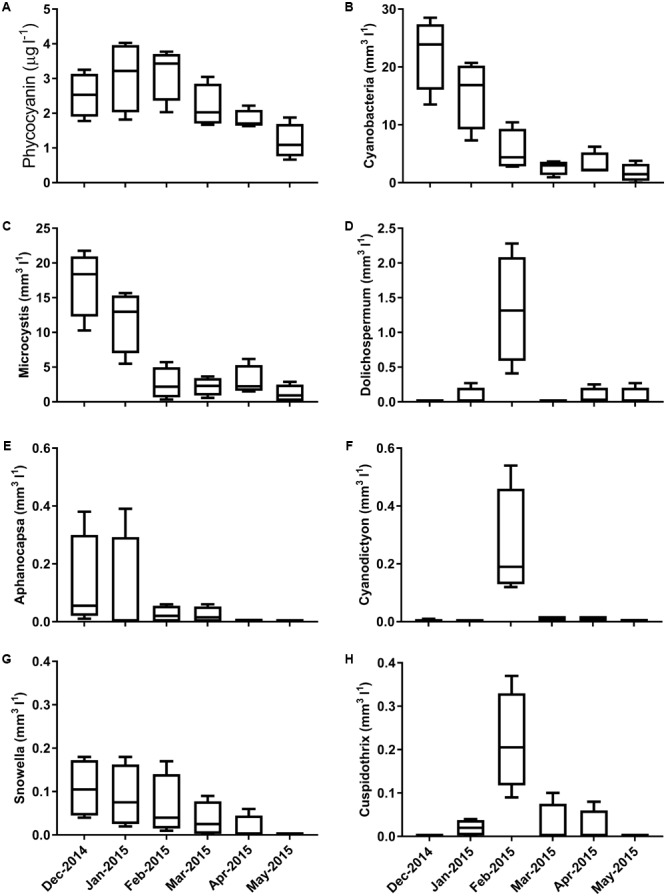
Concentrations of **(A)** phycocyanin and cyanobacterial biovolumes of **(B)** the total cyanobacterial community **(C)** *Microcystis*, **(D)** *Dolichospermum*,**(E)** *Aphanocapsa*, **(F)** *Cyanodictyon*, **(G)** *Snowella*, and **(H)** *Cuspidothrix* species across the 6 month sampling period.

Samples were collected from five sites within Centennial Parklands on six occasions. Sequencing of these samples resulted in the identification of 30,631 OTUs, of which 25,264 were identified as not affiliated with the phylum Cyanobacteria. Analysis of cyanobacterial biovolumes revealed the presence of a large cyanobacterial bloom between December 2014 and February 2015 (**Figure 1**). Biovolumes exceeded 25 mm^3^ l^-1^ (5 × 10^5^cells ml^-1^) in February 2014 before sharply decreasing. The bloom was initially dominated by the cyanobacterial genus *Microcystis* and later by *Dolichospermum*. Other major contributing genera included *Aphanocapsa, Cyanodictyon, Snowella*, and *Cuspidothrix* (**Figure 1**).

PERMANOVA identified both a significant effect of site (Pseudo-F = 1.6001, *p* = 0.005) and date (Pseudo-F = 2.6613, *p* = 0.001) on the microbial community composition. Pairwise *t*-test identified a significant difference of one site from all other sites, independent of date [P_(perm)_ = 0.001–0.005], whereas no significant difference was observed amongst the remaining sites, independent of date [P_(perm)_ = 0.06–0.29]. Following removal of this single site, there was no significant effect of site (Pseudo-F = 1.2334, *p* = 0.1), however, there was a significant effect of date (Pseudo-F = 2.4212, *p* = 0.001). Pairwise each month was significantly different from that preceding it [*t* = 1.33–1.44, P(perm) = 0.002–0.007] with the exception of February 2015 which was not significantly different from the January 2015 sampling [P(perm) = 0.058].

**FIGURE 2:**
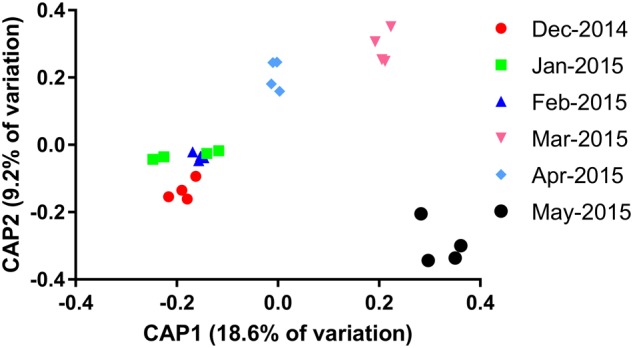
Constrained analyses of principle components (CAP) analysis of the particle-associated microbial community composition constrained by sampling date.

Constrained analyses of principle (CAP) components were implemented in order better visualize the separation of each sampling interval. A significant discrimination of sites was evident for eight components, together explaining 60% of the variation (canonical root = 0.98, *p* = 0.001). The first two axes (**Figure 2**) explained 18.6% and 9.2% of the variation and were able to clearly discriminate between the first three sampling points (December 2014, January 2015, and February 2015), March 2015, April 2015, and May 2015. A smaller discrimination could be seen between the December 2014 sampling and the January/February 2015 sampling, although discrimination of the latter two was not possible, as reflected by the non-significant separation of these samples as demonstrated previously. Species richness and evenness tended to increase as the sampling period progressed, most notably following a decrease in overall cyanobacterial cell numbers in February 2015.

### Stability of the Microbial Community

**FIGURE 3:**
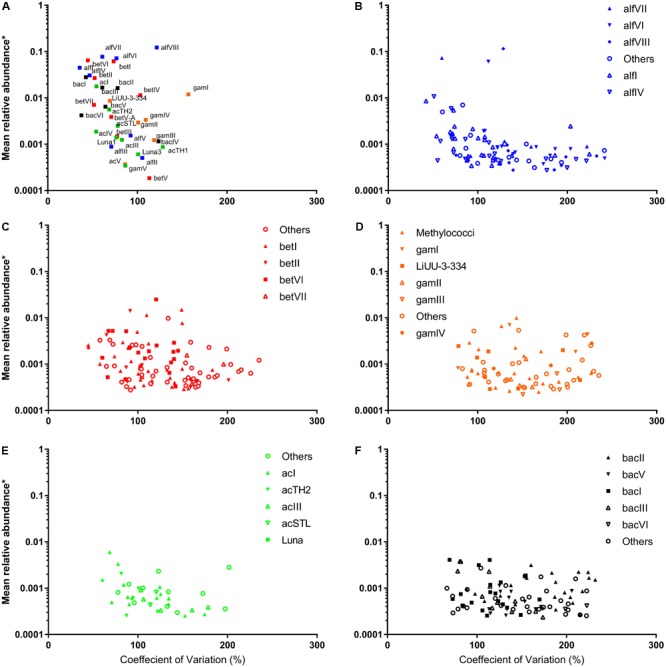
Relative abundance of freshwater **(A)** clades and **(B–F)** OTUs as a function of the coeffecient of variation.

Following selection of OTUs, which exhibited at least 0.1% contribution to any of the 30 sample points, the absolute abundances of 554 OTUs were tested for stability using the CV as a proxy (Linz et al., 2017). Of these 554 OTUs, 54 occurred in a maximum of five samples and so were considered inherently unstable. Of the remaining 500, 46 exhibited a CV value exceeding 200 and were further considered unstable. Stable taxa contributed more to the overall microbial community during stable periods (December–February, **Figure 2**) and decreased as cyanobacterial abundances and cDOM increased (**Supplementary Figure S1**). Stability manifested differently across the major freshwater phyla. As expected, stability was higher at the clade/family level than at the OTU level (**Figure 3**). There was a general trend amongst freshwater clades, whereby those exhibiting a higher relative abundance (log-transformed) tended to exhibit a lower CV value (*r* = -0.401 *p* = 0.014) (**Figure 3A**). Alpha- and Betaproteobacterial clades tended to be abundant and stable (**Figures 3A–C**), whereas Gammaproteobacterial and Actinobacterial clades tended to be of lower abundance (**Figures 3A,D–F**). Two notable exceptions were alfVIII (Alphaproteobacteria) and gamI (Gammaproteobacteria), which were less stable relative to their abundance than expected (**Figure 3A**). At the OTU level, this trend of higher abundance conferring lower CV values was far less evident (**Figures 3B–F**). This was emphasized with many Alphaproteobacterial and Betaproteobacterial OTUs at lower abundance possessing lower CV values than those most abundant OTUs. Generally, at the OTU level, Alphaproteobacteria (mean = 126.5, SD = 49.57) and Betaproteobacteria (mean = 124.6, SD = 42.53) exhibited on average a lower CV value, albeit with higher standard deviation, whereas Gammaproteobacteria (mean = 151.3, *SD* = 44.75) and Bacteroidetes (mean = 145.5, *SD* = 45.44) exhibited higher CV values (**Supplementary Figure S2**). Interestingly, Actinobacterial OTUs (mean = 117.6, *SD* = 35.84), despite their overall lower relative abundance, exhibited consistently lower CV values than other freshwater taxa.

### Network Analyses

Linear correlation analysis was implemented to explore the relationships between the absolute abundance of freshwater genera to environmental variables (pH, temp, DO, etc.), cyanobacterial biovolumes and molecular data. We evaluated the clustering coefficient, degree, and betweenness centrality of each node within the network, with regard to stable vs. unstable OTUs, environmental variables, cyanobacterial cell counts and molecular data. ANOVA revealed a significant difference in degree between these groups in (*F* = 6.22, *p* < 0.001), but not for betweenness (*p* = 0.13) or clustering (*p* = 0.21). Pair-wise, significant differences in degree were observed between stable OTUs, and unstable OTUs (*p* = 0.047). QPCR data including cyanobacterial-specific 16S rRNA copy, *sxt* copy and *mcy* copy, exhibited significantly higher degree scores than OTUs, and cell counts (*p* = 0.001–0.015).

**FIGURE 4:**
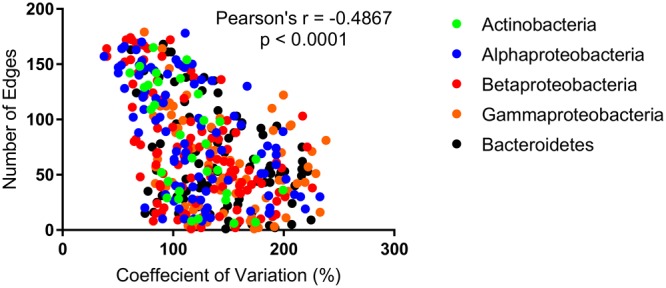
The number of edges (correlations) per OTU as a function of the coefficient of variation (CV).

Within the network 44 of the 46 unstable OTUs, present in greater than six samples, were correlated with other OTUs. Of these, 21 were correlated with environmental parameters, cell counts, or molecular data. Of the 454 stable OTUs, 453 were correlated with other OTUs. Whereas, of these only 246 were correlated with cell counts, molecular data, or environmental data. There was no significant difference (*p* = 0.27) between the average number of correlations between unstable (3.524 ± 0.5921) and stable (4.243 ± 0.1782) and either molecular, environmental, or cell count data. There was, however, a significant difference (*p* < 0.0001) in the average number of correlations exhibited by unstable (47.77 ± 4.209) and stable (74.3 ± 2.284) OTUs with other OTUs. More generally, a linear correlation between the CV and OTU–OTU degree could be observed for all nodes (*r* = -0.0483, *p* < 0.0001) (**Figure 4**), a pattern that was not reproducible for OTU-Environmental/Molecular/Cell Count degree (*r* = -0.086, *p* = 0.146).

**FIGURE 5:**
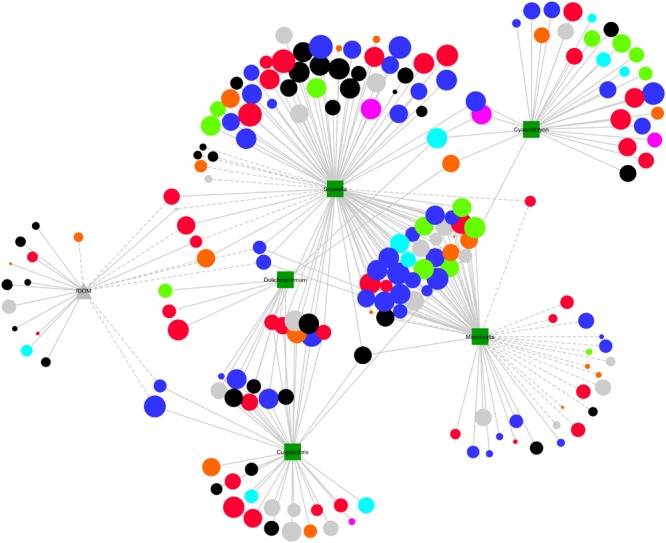
Sub-network visualizing linear correlations between OTUs (circles) and either cyanobacterial biovolumes (squares) or fDOM (triangle). The size of each OTUs is inversely correlated with the CV value for that node, such that larger nodes are considered more stable than smaller ones. Node color represents phylum/class level taxonomies; Actinobacteria (green), Alphaproteobacteria (blue), Betaproteobacteria (red), Gammaproteobacteria (orange), Bacteroidetes (black), others (gray). Solid lines represent positive linear correlations and dashed lines negative linear correlations.

A sub-network of correlations strictly between OTUs and cell counts or fDOM was constructed to visualize the relative contribution of each factor to the occurrence of stable and unstable OTUs (**Figure 5**). The most stable OTUs, largely comprising Alpha- (blue nodes) and Betaproteobacteria (red nodes), were positively correlated with either singularly *Snowella*, which exhibited a largely uniform distribution across the sampling period, or *Snowella* and *Microcystis*. Other cyanobacterial genera, *Cuspidothrix, Cyanodictyon*, and *Dolichospermum*, exhibited exclusively positive correlations with mostly stable OTUs. In regard to unstable OTUs, either *Snowella* or *Microcystis* exhibited mostly negative correlations with unstable OTUs although a number of positive correlations were also present. *Microcystis* exhibited the largest number of negative correlations to both stable and unstable OTUs. fDOM was exclusively positively correlated with unstable OTUs including several unclassified, Bacteroidetes, and Gammaproteobacterial OTUs.

## Discussion

Typically, in aquatic systems, the particle-associated community is considered in its entirety with only moderate consideration given to the differing nature of the particles within the system (Bagatini et al., 2014; Shao et al., 2014; Louati et al., 2015). Different size fractions and even individual particles can harbor distinct microbial communities, with little or no resemblance to the “average” community composition (Brachvogel et al., 2001; Lemarchand et al., 2006; Bižić-Ionescu et al., 2015; Yung et al., 2016). With this in mind, whilst still considering the “bulk” particle associated community, in this study we sought to understand the contribution of temporal variability, specifically changing species composition and influx of terrestrial organic matter, on the bacterial community composition of freshwater cyanobacterial assemblages.

High temporal variability present in the cyanobacterial assemblage (**Figure 1**) was similarly mirrored in the microbial community composition (**Figure 2**). A clear temporal effect was evident across all sampling time points, with the exception of February 2015, which was not significantly different from January 2015. During this period we saw a decrease in total biovolumes, largely driven by *Microcystis* and a proliferation of *Dolichospermum* spp. and to a lesser extent *Cyanodictyon* spp. and *Cuspidothrix* spp. Phycocyanin concentrations, whilst not aligned with total cyanobacterial biovolumes, more closely reflected the changes in microbial community composition, with concentrations exceeding 2 μg l^-1^ characterizing the first 3 months of sampling which were closely clustered (**Figure 2**). In addition to its role in light harvesting phycocyanins may act as storage molecules, with concentrations differing between and within different species (Boussiba and Richmond, 1980). That fluctuations in phycocyanin, rather than species composition (Louati et al., 2015; Woodhouse et al., 2016; Zhu et al., 2016), better reflected changes in community composition, suggests that the microbial community was largely driven by changes in the concentration and quality of the organic matter pool.

We implemented the CV to better understand to what extent changes in species composition and phycocyanin concentrations resulted in changes in the abundance of specific microbial taxa (Linz et al., 2017). The stable occurrence of microbial taxa within the particulome manifested at different taxonomic levels. The CV value only exceeded 150% in the instance of gamI, which is generally linked to the terrestrial environment (Newton et al., 2011; Linz et al., 2017). At the OTU level, this stable occurrence was largely restricted, in each case to a few distinct OTUs, with other OTUs exhibiting far reduced stability. This suggests that a dominant OTU, may have possessed a set of particular metabolic pathways which confer an ecology advantage or simply have been fortunate to be more abundant during the initial stages of particle colonization, restricts the ability of others to establish a presence (Burke et al., 2011). In this context, we considered that this observation could be related to the number of OTUs competing for “space” within their functional clade. “Space” is considered here to be the average phylum abundance. For instance, the high average stable occurrence of the Actinobacteria (**Figure 3** and **Supplementary Figure S2**) is consistent with the low number of OTUs, although the average abundance of these OTUs when present never exceeds 0.01%. Similarly, the average abundance of Bacteroidetes and Gammaproteobacteria never exceed 0.01%, yet these phyla contain many more OTUs. Within this study, compared to the Actinobacteria, the Bacteroidetes and Gammaproteobacteria, have far more OTUs competing for a similar sized “space” and this manifests as lower stability with niche filtering potentially playing a much larger role. The Alpha- and Betaproteobacteria exhibit a compromise with many OTUs but also a greater functional “space”.

Using network analysis, we established whether associations between microorganisms and between microorganisms and their environment, including cyanobacterial species drive their stable occurrence. We first established unstable OTUs as any OTU with a CV value greater than 200, a value that is ultimately arbitrary, given that the CV is clearly a continuum (**Figures 3, 4** and **Supplementary Figure S2**; Linz et al., 2017). We considered the contribution of each node to the network structure using three metrics, degree (number of edges), clustering coefficient (representing the number of edges possessed by first neighbors) and the betweenness centrality (representing the number of times a node is present on the shortest path between any two nodes). Betweenness and clustering are typically considered variables representing modularity of networks and nodes with high values are often considered hub or key-stone species (Eldridge et al., 2015; Guidi et al., 2016). Generally, whilst there were no significant differences between betweenness and clustering of stable vs. unstable nodes, nodes considered stable possessed the highest values and were similar in this regard to environmental variables including pH and temperature (Woodhouse et al., 2016). A significant difference was observed between the degree of stable vs. unstable OTUs suggesting that stable OTUs are more connected to other OTUs, environmental data and cyanobacterial biovolumes. When we further scrutinized this relationship we found that this was not the case for the number of connections between stable and unstable OTUs and cyanobacterial biovolumes, environmental and qPCR data. For the most part cyanobacteria were positively correlated with stable rather than unstable OTUs. *Microcystis* was an exception exhibiting a large number of both positive and negative correlations across the stability continuum (**Figure 5**). Several studies have previously shown both advantageous and deleterious effects of *Microcystis* exudates, particularly microcystin, on heterotrophic microorganisms (Casamatta and Wickstrom, 2000; Berg et al., 2009; Mou et al., 2013). Most critically, deleterious effects were more common where *Microcystis* blooms were infrequent or non-existent (Casamatta and Wickstrom, 2000). The negative correlations between *Microcystis* and stable OTUs may reflect this phenomenon, although *Microcystis* spp. blooms in Kensington Pond are commonplace. Furthermore, positive correlations between *Microcystis* and unstable OTUs may reflect the ability of these heterotrophic bacteria to take advantage of the intermittent release of microcystin (Mou et al., 2013; Lezcano et al., 2017). However, organisms typically affiliated with microcystin degradation [*Sphingomonas* (alfIII), *Sphingophyxis* (alfIV)] were not correlated with *Microcystis* spp. (Lezcano et al., 2017). Together, this suggests that whilst environmental partitioning and cyanobacterial species abundance influence the abundance of individual OTUs (**Figure 5**), it does not influence average overall stability of the microbial community.

More importantly, however, was the significant difference between the number of OTU–OTU correlations for stable and non-stable OTUs and the strong linear correlation that was observed between degree and CV. This relationship was also apparent within each freshwater phyla, albeit to a different extent. Individually, the Actinobacteria, Alphaproteobacteria, and Betaproteobacteria exhibited relationships stronger than the average, whilst the Gammaproteobacteria and Bacteroidetes were weaker (**Figure 4**). This suggests that the stable occurrence of heterotrophic bacteria is strongly related to the associations, both negative and positive, with other heterotrophic bacteria. Furthermore, that this relationship manifests differently across taxonomic groups may reflect that some groups are more dependent than others on cross-feeding for meeting nutrient requirements (e.g., Seth and Taga, 2014).

Heavy rainfall and clearance of vegetation around the catchment between the April–May sampling periods resulted in visibly large inputs of allochthonous organic matter coincided with a decrease in the abundance of cyanobacterial biomass and an increase in the fDOM. Fewer OTUs were directly correlated with fDOM than with factors associated with cyanobacterial biomass and were restricted to OTUs affiliated with the Bacteroidetes and Gammaproteobacteria, which were on average less stable. This stable occurrence likely reflects the weak affinity of these groups to specialize in freshwater environments, with the Gammaproteobacteria demonstrating a stronger affinity for terrestrial systems (Newton et al., 2011). Overall, fewer stable OTUs were dominant during periods of high fDOM, although establishing their true stability is limited given the number of sampling points covering this period. The low abundance of stable OTUs during this period was consistent with an increase in species richness, despite a decrease in algal abundance. This is consistent with the concept, previously proposed, that in the absence of a few dominant niche organisms are less stable and more likely to be specialized, resulting in higher species richness.

In summary, we found that cyanobacterial associated microbial assemblages are comprised largely of stable dominant taxa. A large number of correlations between heterotrophic taxa were associated with this increased stability, which we speculate is due to a high inter-dependency. The notion that high connectivity within microbial and other ecological networks restricts invasion of novel species is supported by a strong relationship between degree and CV. The stable occurrence of particular microorganisms in this regard is not always a net positive in regards to ecosystem functioning and biodiversity. High connectivity amongst microbial communities in arid soils has been related to a lack of permissibility in nutrient uptake and a contributing factor to low spatial heterogeneity (Eldridge et al., 2015). Several studies have highlighted the importance of microbial diversity in driving ecosystem functionality. In particular in aquatic systems microbial diversity is an important driver of broad (respiration) and specialized (toxin-degradation) microbial processes (Delgado-Baquerizo et al., 2016). However, in both terrestrial and aquatic ecosystems stability, productivity and diversity in function are not always mutual propositions. Productive periods are often highly unstable, stable lake ecosystems not always productive; and functional diversity is selected against, by invasion of opportunistic organisms, during periods of highly labile organic matter. Here, we have provided evidence to suggest that microbial communities associated with cyanobacterial blooms are inherently stable, however, we also stress that inputs of allochthonous organic matter and associated microbes derived from increased terrestrial run-off have the potential to disrupt this stability.

## Author Contributions

JW and BN designed the experimental work and sampling regime. JW and JZ performed field sampling and generated data. JW, JZ, and H-PG analyzed the data. JW, JZ, H-PG, and BN prepared the manuscript.

## Conflict of Interest Statement

The authors declare that the research was conducted in the absence of any commercial or financial relationships that could be construed as a potential conflict of interest.

## Footnotes

1 https://github.com/mcmahon-uw/FWMFG

2 https://github.com/McMahonLab/TaxAss

## Supplementary Material

FIGURE S1 Relative contribution of stable (CV > 200%) OTUs to the overall community composition.

FIGURE S2 Distribution of CV values as a factor of phylum/class.

## References

[B1] Adam B. Klawonn I. Svedén J. B. Bergkvist J. Nahar N. Walve J. (2016). N2-fixation, ammonium release and N-transfer to the microbial and classical food web within a plankton community. *ISME J.* 10 450–459. 10.1038/ismej.2015.126 26262817 PMC4737936

[B2] Amemiya Y. Kato K. Okino T. Nakayama O. (1990). Changes in the chemical composition of carbohydrates and proteins in surface water during a bloom ofMicrocystis in Lake Suwa. *Ecol. Res.* 5 153–162. 10.1007/BF02346987

[B3] Azam F. Fenchel T. Field J. G. Gray J. S. Meyer-Reil L. A. Thingstad F. (1983). The ecological role of water-column microbes in the sea. *Mar. Ecol. Prog. Ser.* 10 257–263. 10.3354/meps010257

[B4] Bagatini I. L. Eiler A. Bertilsson S. Klaveness D. Tessarolli L. P. Vieira A. A. H. (2014). Host-specificity and dynamics in bacterial communities associated with bloom-forming freshwater phytoplankton. *PLoS One* 9:e85950. 10.1371/journal.pone.0085950 24465807 PMC3896425

[B5] Bell W. Mitchell R. (1972). Chemotactic and growth responses of marine bacteria to algal extracellular products. *Biol. Bull.* 143 265–277. 10.2307/1540052

[B6] Berg K. A. Lyra C. Sivonen K. Paulin L. Suomalainen S. Tuomi P. (2009). High diversity of cultivable heterotrophic bacteria in association with cyanobacterial water blooms. *ISME J.* 3 314–325. 10.1038/ismej.2008.110 19020559

[B7] Bižić-Ionescu M. Zeder M. Ionescu D. Orlić S. Fuchs B. M. Grossart H. P. (2015). Comparison of bacterial communities on limnic versus coastal marine particles reveals profound differences in colonization. *Environ. Microbiol.* 17 3500–3514. 10.1111/1462-2920.12466 24674021

[B8] Boussiba S. Richmond A. E. (1980). C-phycocyanin as a storage protein in the blue-green alga *Spirulina platensis*. *Arch. Microbiol.* 125 143–147. 10.1007/BF00403211

[B9] Bouvy M. Pagano M. Troussellier M. (2001). Effects of a cyanobacterial bloom (*Cylindrospermopsis raciborskii*) on bacteria and zooplankton communities in Ingazeira reservoir (northeast Brazil). *Aquat. Microb. Ecol.* 25 215–227. 10.3354/ame025215

[B10] Brachvogel T. Schweitzer B. Simon M. (2001). Dynamics and bacterial colonization of microaggregates in a large mesotrophic lake. *Aquat. Microb. Ecol.* 26 23–35. 10.3354/ame026023

[B11] Briand E. Humbert J. F. Tambosco K. Bormans M. Gerwick W. H. (2016). Role of bacteria in the production and degradation of *Microcystis* cyanopeptides. *MicrobiologyOpen* 5 469–478. 10.1002/mbo3.343 26918405 PMC4905998

[B12] Burke C. Thomas T. Lewis M. Steinberg P. Kjelleberg S. (2011). Composition, uniqueness and variability of the epiphytic bacterial community of the green alga *Ulva australis*. *ISME J.* 5 590–600. 10.1038/ismej.2010.164 21048801 PMC3105733

[B13] Carey C. C. Ibelings B. W. Hoffmann E. P. Hamilton D. P. Brookes J. D. (2011). Eco-physiological adaptations that favour freshwater cyanobacteria in a changing climate. *Water Res.* 46 1394–1407. 10.1016/j.watres.2011.12.016 22217430

[B14] Caron D. A. Goldman J. C. Dennett M. R. (1986). Effect of temperature on growth, respiration and nutrient regeneration by an omnivorous microflagellate. *Appl. Environ. Microbiol.* 52 1340–1347. 16347239 10.1128/aem.52.6.1340-1347.1986 PMC239231

[B15] Casamatta D. A. Wickstrom C. E. (2000). Sensitivity of two disjunct bacterioplankton communities to exudates from the cyanobacterium *Microcystis aeruginosa* Kützing. *Microb. Ecol.* 40 64–73. 10.1007/s002480000035 10977878

[B16] Clarke K. R. Warwick R. M. (2005). *Primer-6 Computer Program.* Plymouth: Natural Environment Research.

[B17] Crawford A. Holliday J. Merrick C. Brayan J. van Asten M. Bowling L. (2017). Use of three monitoring approaches to manage a major *Chrysosporum ovalisporum* bloom in the Murray River, Australia, 2016. *Environ. Monit. Assess.* 189:202. 10.1007/s10661-017-5916-4 28364328

[B18] Delgado-Baquerizo M. Giaramida L. Reich P. B. Khachane A. N. Hamonts K. Edwards C. (2016). Lack of functional redundancy in the relationship between microbial diversity and ecosystem functioning. *J. Ecol.* 104 936–946. 10.1111/1365-2745.12585

[B19] Dziallas C. Grossart H. P. (2011). Temperature and biotic factors influence bacterial communities associated with the cyanobacterium *Microcystis* sp. *Environ. Microbiol.* 13 1632–1641. 10.1111/j.1462-2920.2011.02479.x 21492362

[B20] Dziallas C. Grossart H. P. (2012). Microbial interactions with the cyanobacterium *Microcystis aeruginosa* and their dependence on temperature. *Mar. Biol.* 159 2389–2398. 10.1007/s00227-012-1927-4

[B21] Eiler A. Bertilsson S. (2004). Composition of freshwater bacterial communities associated with cyanobacterial blooms in four Swedish lakes. *Environ. Microbiol.* 6 1228–1243. 10.1111/j.1462-2920.2004.00657.x 15560821

[B22] Eiler A. Heinrich F. Bertilsson S. (2012). Coherent dynamics and association networks among lake bacterioplankton taxa. *ISME J.* 6 330–342. 10.1038/ismej.2011.113 21881616 PMC3260505

[B23] Eldridge D. J. Woodhouse J. N. Curlevski N. J. Hayward M. Brown M. V. Neilan B. A. (2015). Soil-foraging animals alter the composition and co-occurrence of microbial communities in a desert shrubland. *ISME J.* 9 2671–2681. 10.1038/ismej.2015.70 25932616 PMC4817632

[B24] Grossart H. P. (2010). Ecological consequences of bacterioplankton lifestyles: changes in concepts are need. *Environ. Microbiol. Rep.* 2 706–714. 10.1111/j.1758-2229.2010.00179.x 23766274

[B25] Guidi L. Chaffron S. Bittner L. Eveillard D. Larhlimi A. Roux S. (2016). Plankton networks driving carbon export in the oligotrophic ocean. *Nature* 532 465–470. 10.1038/nature16942 26863193 PMC4851848

[B26] Havens K. E. (2008). “Cyanobacterial blooms: effects on aquatic ecosystems,” in *Cyanobacterial Harmful Algal Blooms: State of Science and Research Needs* ed. Hudnell H. K. (Berlin: Springer) 733–747. 10.1007/978-0-387-75865-7_33 18461790

[B27] Hötzel G. Croome R. (1999). *A Phytoplankton Methods Manual for Australian Freshwaters.* Canberra: Land and Water Resources, Research and Development Corporation.

[B28] Kehr J. C. Dittmann E. (2015). Biosynthesis and function of extracellular glycans in cyanobacteria. *Life* 5 164–180. 10.3390/life5010164 25587674 PMC4390846

[B29] Klawonn I. Bonaglia S. Brüchert V. Ploug H. (2015). Aerobic and anaerobic nitrogen transformation processes in N2-fixing cyanobacterial aggregates. *ISME J.* 9 1456–1466. 10.1038/ismej.2014.232 25575306 PMC4438332

[B30] Kolmonen E. Sivonen K. Rapala J. Haukka K. (2004). Diversity of cyanobacteria and heterotrophic bacteria in cyanobacterial blooms in Lake Joutikas, Finland. *Aquat. Microbiol. Ecol.* 36 201–211. 10.3354/ame036201 19020559

[B31] Lemarchand C. Jardillier L. Carrias J. F. Richardot M. Debroas D. Sime-Ngando T. (2006). Community composition and activity of prokaryotes associated to detrital particles in two contrasting lake ecosystems. *FEMS Microbiol. Ecol.* 57 442–451. 10.1111/j.1574-6941.2006.00131.x 16907758

[B32] Lezcano M. Á. Velázquez D. Quesada A. El-Shehawy R. (2017). Diversity and temporal shifts of the bacterial community associated with a toxic cyanobacterial bloom: an interplay between microcystin producers and degraders. *Water Res.* 125 52–61. 10.1016/j.watres.2017.08.025 28829999

[B33] Linz A. M. Crary B. C. Shade A. Owens S. Gilbert J. A. Knight R. (2017). Bacterial community composition and dynamics spanning five years in freshwater bog lakes. *mSphere* 2:e00169-17. 10.1128/mSphere.00169-17 28680968 PMC5489657

[B34] Louati I. Pascault N. Debroas D. Bernard C. Humbert J. F. Leloup J. (2015). Structural diversity of bacterial communities associated with bloom-forming freshwater cyanobacteria differs according to the cyanobacterial genus. *PLoS One* 10:e0140614. 10.1371/journal.pone.0140614 26579722 PMC4651346

[B35] Morin N. Vallaeys T. Hendrickx L. Natalie L. Wilmotte A. (2010). An efficient DNA isolation protocol for filamentous cyanobacteria of the genus *Arthrospira*. *J. Microbiol. Methods* 80 148–154. 10.1016/j.mimet.2009.11.012 20004220

[B36] Mou X. Lu X. Jacob J. Sun S. Heath R. (2013). Metagenomic identification of bacterioplankton taxa and pathways involved in microcystin degradation in Lake Erie. *PLoS One* 8:e61890. 10.1371/journal.pone.0061890 23637924 PMC3634838

[B37] Newcombe G. (ed.) (2009). *International Guidance Manual for the Management of Toxic Cyanobacteria.* London: Global Water Research Coalition.

[B38] Newton R. J. Jones S. E. Eiler A. McMahon K. D. Bertilsson S. (2011). A guide to the natural history of freshwater lake bacteria. *Microbiol. Mol. Biol. Rev.* 75 14–49. 10.1128/MMBR.00028-10 21372319 PMC3063352

[B39] Paerl H. W. (2000). “Marine plankton,” in *The Ecology of Cyanobacteria: Their Diversity in Time and Space* eds Whitton B. A. Potts M. (Dordrecht: Kluwer Academic Publishers) 121–148.

[B40] Peura S. Bertilsson S. Jones R. I. Eiler A. (2015). Resistant microbial cooccurrence patterns inferred by network topology. *Appl. Environ. Microbiol.* 81 2090–2097. 10.1128/AEM.03660-14 25576616 PMC4345367

[B41] Pomeroy L. R. Wiebe W. J. (1988). Energetics of microbial food webs. *Hydrobiologia* 159 7–18. 10.1007/BF00007363 11334303

[B42] Pomeroy L. R. Wiebe W. J. Deibel D. Thompson R. J. Rowe G. T. Pakulski J. D. (1991). Bacterial responses to temperature and substrate concentration during the Newfoundland spring bloom. *Mar. Ecol. Prog. Ser.* 75 143–159. 10.3354/meps075143

[B43] Porter K. G. Feig Y. S. (1980). The use of DAPI for identifying and counting aquatic microflora. *Limnol. Oceanogr.* 25 943–948. 10.4319/lo.1980.25.5.0943

[B44] Salomon P. S. Janson S. Granéli E. (2003). Molecular identification of bacteria associated with filaments of *Nodularia spumigena* and their effect on the cyano-bacterial growth. *Harmful Algae* 2 261–272. 10.1016/S1568-9883(03)00045-3

[B45] Schloss P. D. Westcott S. L. (2011). Assessing and improving methods used in operational taxonomic unit-based approaches for 16S rRNA gene sequence analysis. *Appl. Environ. Microbiol.* 77 3219–3226. 10.1128/AEM.02810-10 21421784 PMC3126452

[B46] Schloss P. D. Westcott S. L. Ryabin T. Hall J. R. Hartmann M. Hollister E. B. (2009). Introducing mothur: open-source, platform-independent, community-supported software for describing and comparing microbial communities. *Appl. Environ. Microbiol.* 75 7537–7541. 10.1128/AEM.01541-09 19801464 PMC2786419

[B47] Seth E. C. Taga M. E. (2014). Nutrient cross-feeding in the microbial world. *Front. Microbiol.* 5:530. 10.3389/fmicb.2014.00350 25071756 PMC4086397

[B48] Shannon P. Markiel A. Ozier O. Baliga N. S. Wang J. T. Ramage D. (2003). Cytoscape: a software environment for integrated models of biomolecular interaction networks. *Genome Res.* 13 2498–2504. 10.1101/gr.1239303 14597658 PMC403769

[B49] Shao J. Jiang Y. Wang Z. Peng L. Luo S. Gu J. (2014). Interactions between algicidal bacteria and the cyanobacterium *Microcystis aeruginosa*: lytic characteristics and physiological responses in the cyanobacteria. *Int. J. Environ. Sci. Technol.* 11 469–476. 10.1007/s13762-013-0205-4

[B50] Sivonen K. Börner T. (2008). “Bioactive compounds produced by cyanobacteria,” in *The Cyanobacteria: Molecular Biology, Genomics and Evolution* eds Herrero A. Flores E. (Norfolk: Caister Academic Press) 159–197.

[B51] Smoot M. E. Ono K. Ruscheinski J. Wang P. L. Ideker T. (2011). Cytoscape 2.8: new features for data integration and network visualization. *Bioinformatics* 27 431–432. 21149340 10.1093/bioinformatics/btq675 PMC3031041

[B52] Tonietto A. E. Lombardi A. T. Vieira A. A. H. Parrish C. C. Choueri R. B. (2014). *Cylindrospermopsis raciborskii* (Cyanobacteria) exudates: chemical characterization and complexation capacity for Cu, Zn, Cd and Pb. *Water Res.* 49 381–390. 10.1016/j.watres.2013.10.025 24169513

[B53] Tuomainen J. Hietanen S. Kuparinen J. Martikainen P. J. Servomaa K. (2006). Community structure of the bacteria associated with *Nodularia* sp. (cyanobacteria) aggregates in the Baltic Sea. *Microb. Ecol.* 53 513–522. 10.1007/s00248-006-9130-0 16944338

[B54] Wikner J. Rassoulzadegan F. Hagtröm Å (1990). Periodic bacterivore activity balances bacterial growth in the marine environment. *Limnol. Oceanogr.* 35 313–324. 10.4319/lo.1990.35.2.0313

[B55] Wilson A. E. Sarnelle O. Tillmans A. R. (2006). Effects of cyanobacterial toxicity and morphology on the population growth of freshwater zooplankton: meta-analyses of laboratory experiments. *Limnol. Oceanogr.* 51 1915–1924. 10.4319/lo.2006.51.4.1915

[B56] Woodhouse J. N. Kinsela A. S. Collins R. N. Bowling L. C. Honeyman G. L. Holliday J. K. (2016). Microbial communities reflect temporal changes in cyanobacterial composition in a shallow ephemeral freshwater lake. *ISME J.* 10 1337–1351. 10.1038/ismej.2015.218 26636552 PMC5029192

[B57] Yung C. M. Ward C. S. Davis K. M. Johnson Z. I. Hunt D. E. (2016). Insensitivity of diverse and temporally variable particle-associated microbial communities to bulk seawater environmental parameters. *Appl. Environ. Microbiol.* 82 3431–3437. 10.1128/AEM.00395-16 27037125 PMC4959251

[B58] Zhu L. Zancarini A. Louati I. De Cesare S. Duval C. Tambosco K. (2016). Bacterial communities associated with four cyanobacterial genera display structural and functional differences: evidence from an experimental approach. *Front. Microbiol.* 7:1662. 10.3389/fmicb.2016.01662 27822204 PMC5076464

