# Supplementary figures and images for: Cyanobacterial Community Composition and Bacteria–Bacteria Interactions Promote the Stable Occurrence of Particle-Associated Bacteria
Source: Front Microbiol. 2018 Apr 26;9:777. doi: 10.3389/fmicb.2018.00777 (PMC5932394; doi:10.3389/fmicb.2018.00777)

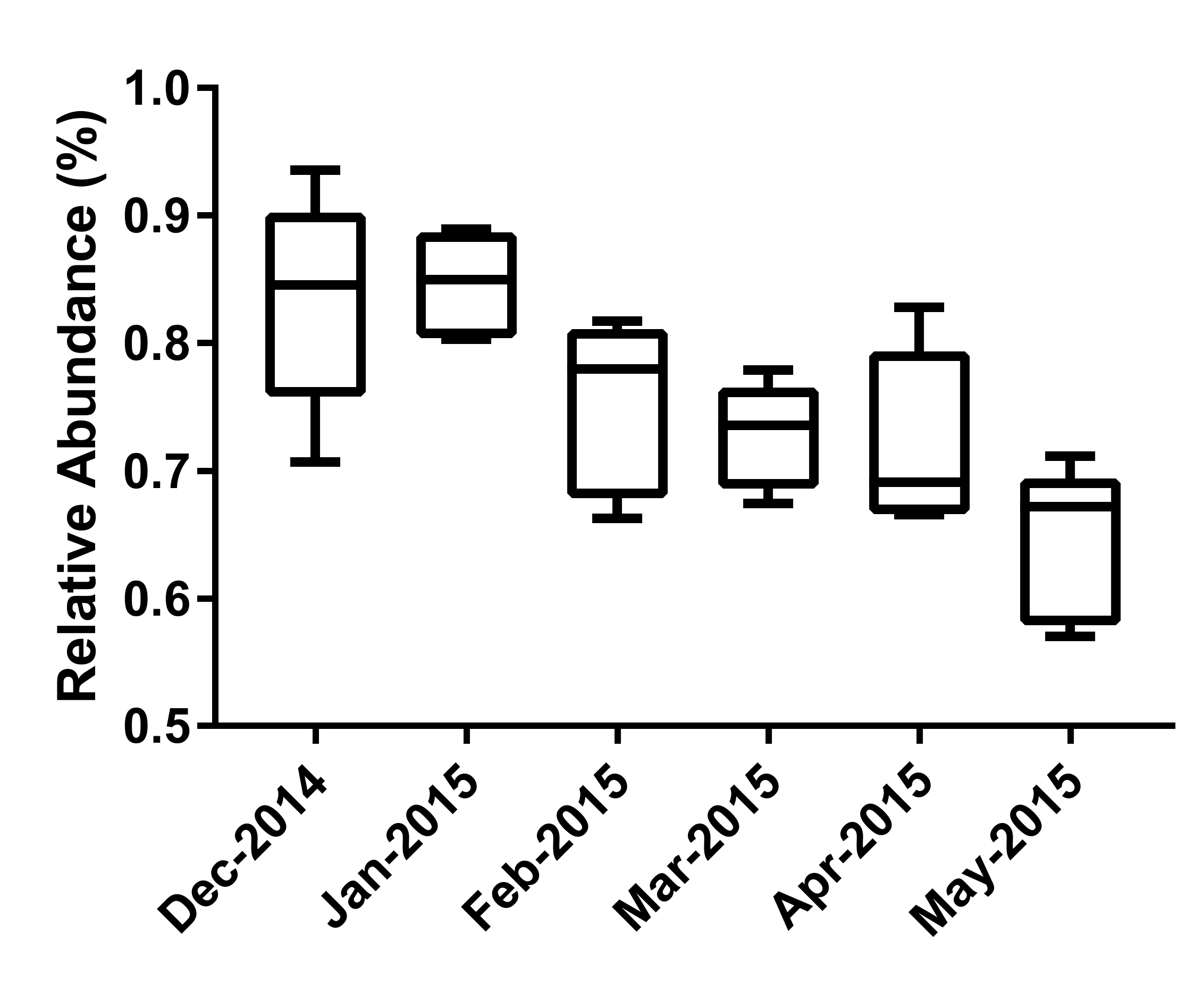

Supplement: FIGURE S1 — Relative contribution of stable (CV > 200%) OTUs to the overall community composition. [file Image_1.TIFF]

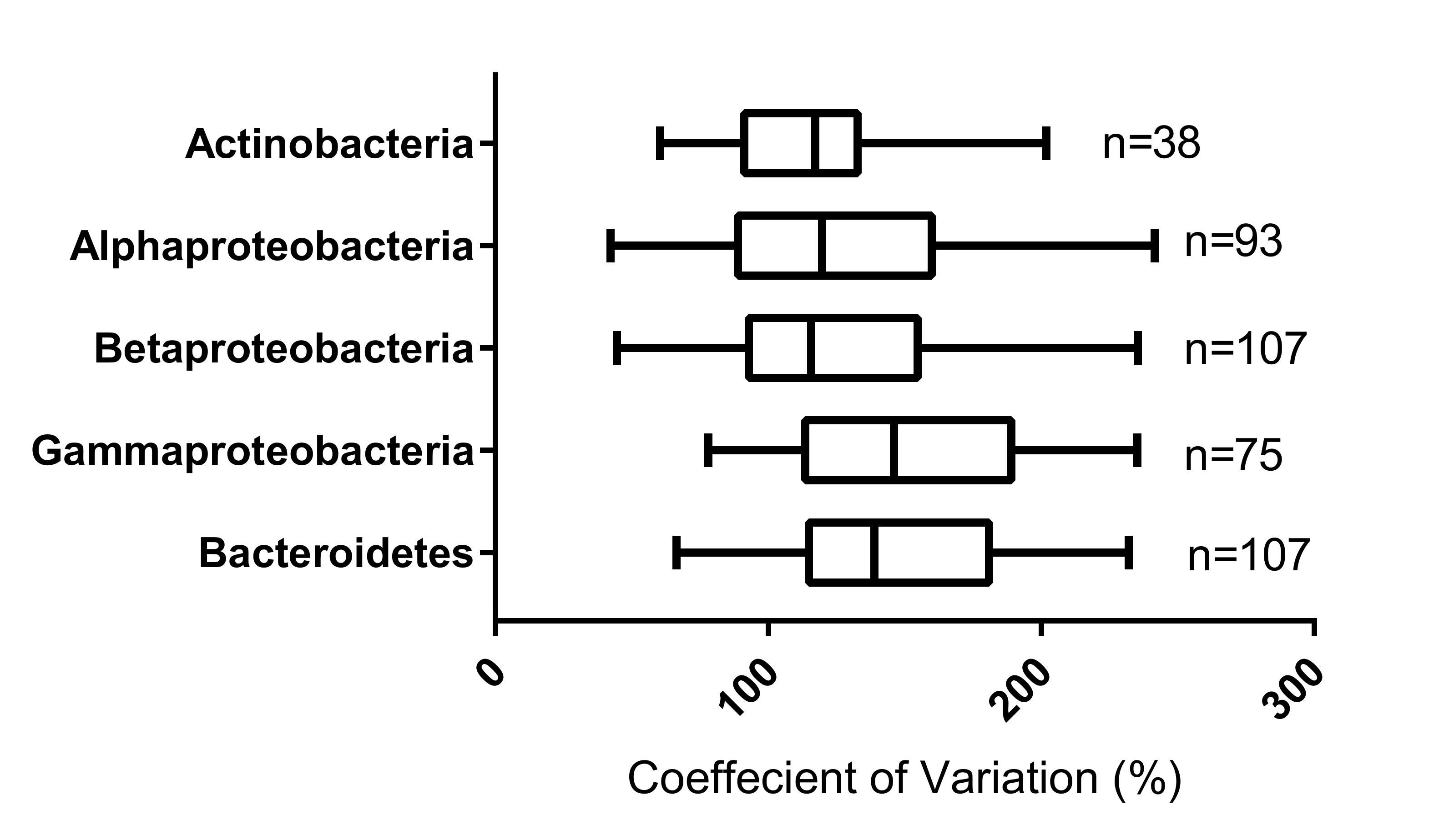

Supplement: FIGURE S2 — Distribution of CV values as a factor of phylum/class. [file Image_2.TIFF]
